# Experimental and CFD Investigation of inlet fin influence on compressor stability and performance

**DOI:** 10.1038/s41598-026-55058-y

**Published:** 2026-05-27

**Authors:** Tariq Ullah, Krzysztof Kantyka, Grzegorz Wasilewski, Krzysztof Sobczak, Grzegorz Liskiewicz

**Affiliations:** 1 Faculty of Mechanical Engineering, Institute of Turbomachinery, Lodz University of Technology, Lodz, 90-924 Poland; 2 Department of Automation, Biomechanics and Mechatronics, Lodz University of Technology, 1/15 Stefanowski St, Lodz, 90-537 Poland

**Keywords:** Centrifugal compressor, Inlet fins, Surge margin, Inlet recirculation, CFD, Energy science and technology, Engineering

## Abstract

The operational stability and overall performance of turbocharger compressors are influenced by local as well as global instability phenomena under off-design conditions. This paper investigates the suppression of inlet recirculation and enhancement of surge operating range, using inlet fins. Both experiments and high-fidelity CFD simulations were used to assess the effect of fins on compressor performance, stability, and operating range. Experiments were conducted on a small turbocharger, while numerical simulations were performed using the RANS equations coupled with the k–ω SST turbulence model solved by ANSYS CFX. The numerical approach was validated, and a mesh independence study was conducted to evaluate discretization uncertainty at both design and off-design conditions. The results revealed that fins effectively suppressed upstream recirculation and preswirl, and promoted more uniform and directed flow into the impeller. Under stable, high-flow conditions, fins had a negligible influence on pressure ratio and efficiency. However, at low flow rates near surge, their presence significantly enhanced compressor performance. Pressure ratio increased by up to 5.4%, while the surge mass flow rate decreased by 8.4–10.4%, indicating a substantial extension of the stable operating range. Despite an efficiency penalty of up to 5% at low flow rates, the compressor performance at medium and high flow rates remains unaffected. The flow field analyses revealed that fins reduced swirl velocity, maintained higher incidence angles, and limited upstream momentum exchange, thereby stabilizing near-surge operation.

## Introduction

Centrifugal compressors are widely used in various applications, including aeronautics, industrial and micro-gas turbine plants, automotive turbochargers, and various process installations. In the automotive sector, turbocharging internal combustion engines (ICEs) is a key technology for improving power-to-weight ratios and efficiency, enabling engine downsizing to meet stricter emission policies and reduce fuel consumption^1^. Further advancements, such as hydrogen combustion and electric turbochargers (e-turbos), promise to enhance sustainability by reducing emissions and improving performance^2^. Over recent decades, the expanding applications and persistent need for enhanced performance have encouraged a significant increase in centrifugal compressor research. With the target of a lower environmental impact, increasingly higher-pressure ratios and efficiency are required. A further design objective is to achieve a wider operating range while maintaining high efficiency, which is critical for systems that frequently operate under off-design conditions. A significant challenge in achieving this extended range is the compressor operation at low mass flow rates, where it becomes susceptible to dangerous flow instabilities such as surge, rotating stall, and inlet recirculation (IR). Surge induces strong fluctuations in the flow structure that can cause damage to the compressor and to the entire system; therefore, this condition must be avoided. Also, local instabilities like rotating stall and inlet recirculation can significantly degrade compressor performance^3^, therefore, their understanding and development of the methods of their mitigation is still a field of active research.

### Local instability of inlet recirculation

The inlet recirculation phenomenon occurs at low flow rates. It is believed to be mostly axisymmetric and creates a ring of blockage around the entire annulus of the rotor inlet, affecting all blade passages. It occurs in various machines, especially radial compressors and pumps, which typically happens when a machine is throttled to low flow conditions. It could be described as an axisymmetric stall, as it produces smaller mass flow fluctuations, allowing stable operation. In contrast, rotating stall generates stronger fluctuations and stresses, posing greater risks to compressor integrity^4^. If a compressor with IR is throttled to even lower flow rates, it will eventually surge, like a compressor experiencing a rotating stall^5^. Knowledge regarding the IR phenomenon and its initiating mechanisms is still unknown. Most of the knowledge comes from blowers and hydraulic pumps. The term churning loss was first used by Daugherty^6^ when he observed IR in centrifugal pumps. Stepanoff^7^ was the first to quantify the loss related to IR in a centrifugal pump. Gulich^8^ provided a comprehensive explanation of IR in hydraulic pumps. Tanaka^9^ conducted experimental research on semi-axial pumps, which provides further examples of IR in incompressible machines. In another study, Muggli et al.^10^ conducted full-annulus numerical simulations on a vertical semi-axial mixed-flow pump and validated the results using casing-mounted pressure measurements. Their analysis revealed the presence of IR and demonstrated that incorporating an inlet rib effectively mitigated this phenomenon.

Inlet recirculation has also been observed in centrifugal compressors. Andersen et al.^11^ documented the progression of IR in a turbocharger impeller using thermocouples positioned at multiple axial locations along the inlet duct. A more detailed classification of compressor instabilities was later provided by Ribaud^12^, who performed experimental investigations on three high-pressure ratio centrifugal compressors and distinguished three rotor operating regimes. At low mass-flow conditions, he identified two distinct forms of recirculation, one occurring at the rotor inlet and another at the rotor exit. In another study, Kaemmerer and Rautenberg^5^ examined a centrifugal compressor equipped with a vaneless diffuser to assess flow behavior across different rotational speeds along the surge line. Their results revealed three stall types: a non-periodic stall associated with flow recirculation at the impeller inlet, and two periodic stall modes emerging at higher speeds. The non-periodic stall observed at low speeds, characterized by flow recirculation, can be interpreted as a form of IR.

To facilitate early-stage compressor design, Qiu et al.^13^ developed a mean line calculation-based recirculation model and validated it using both CFD simulations and experimental measurements. Harley et al.^14^ later refined this model and investigated IR in turbocharger compressors through combined experimental testing and steady-state numerical analysis. Their approach treated recirculation as an effective area blockage, partitioning the inlet into an active flow region and a blocked recirculation region. The study demonstrated that the presence of IR reduces the incidence angle within the active flow zone. Qiu reported that omitting a recirculation loss model results in an unrealistic drop in predicted power at low flow rates, contrary to the increasing trend observed in experimental data. Moreover, efficiency predictions without accounting for recirculation were significantly overestimated when compared to measured efficiencies under low-flow conditions. Qiu further stated that inlet flow recirculation can induce several adverse effects, including flow instability and acoustic emissions^13^.

The presence of IR was further verified through both experimental measurements and numerical simulations by Tamaki et al.^15,16^, who concluded that IR is an unsteady flow feature characterized by broadband pressure fluctuations in the frequency domain. Building on this understanding, Lin et al.^17^ conducted combined numerical and experimental analyses to evaluate the influence of IR on compressor performance. Their results showed a distinct frequency hump in the pressure spectra when the recirculating vortex reached the impeller inlet. Lin also observed that IR initially produced beneficial effects such as reducing incidence, tip-clearance losses, and blade-loading losses, thereby improving compressor performance. However, as the flow rate decreased and IR intensified, its detrimental impacts, including pre-swirl and pre-heating, became dominant and ultimately degraded overall compressor performance. In a related investigation, Schreiber^4^ measured casing-wall pressures upstream of the rotor and complemented the data with CFD simulations. His findings revealed a pronounced recirculation bubble, indicated by all upstream casing pressure taps showing values higher than the inlet total pressure. Additionally, Kulak et al.^18^ examined the spatial evolution of recirculation regions throughout the full surge cycle using unsteady simulations. They reported that the inlet recirculation structure evolved into a three-dimensional toroidal form. In our previous study^19^, IR was characterized as a non-axisymmetric annular region that expanded significantly in both radial and streamwise directions, occupying up to 27% of the inlet area and causing a 15.7% efficiency drop. Its intensity varied circumferentially, reaching a maximum near the volute tongue.

### Inlet recirculation control methods

Modern automotive centrifugal compressors require a wide operating range or an expanded map width, particularly at higher pressure ratios, to achieve greater boost pressure during low-end torque operation. Enhancing efficiency near the surge line is essential for reducing emissions, improving specific fuel consumption, and refining transient response. A pressure ratio curve that steadily increases toward surge (i.e., with a negative gradient) supports stable operation and improved boost at lower speeds. To extend the compressor operating range and prevent stalls or surges, two main flow control strategies are used: passive and active. Active flow control methods require additional energy but allow for real-time and unsteady flow regulation, offering higher effectiveness than passive techniques^20^. However, their reliance on external power, complex controls, and higher maintenance requirements can reduce system reliability and complicate integration into existing designs. Several researchers, Epstein et al.^21^, Pinsley et al.^22^, Ashrafi et al.^23^, Paduano et al.^24^, and D’Andrea et al.^25^ have investigated their use to extend the compressor operating range.

In contrast, passive flow control methods require no external energy and are simpler to implement; however, they cannot be adjusted once in operation. They involve geometric modifications, such as adding inlet fins, grooves, slots, or holes to the compressor casing, to alter internal flow structures. These designs were initially developed through experiments and later refined using one-dimensional modeling^26^. Typically, grooves are positioned near the impeller inducer^27^, though some studies place them adjacent to the blade tip^28^. Several other studies have explored techniques to expand the compressor operating range by modifying the recirculation pattern and mitigating flow instabilities. Tamaki^29^ compared three inlet channel configurations: an upstream slot, a bleed slot, and an annular cavity equipped with vanes that generated a counter-swirl in the intake flow, and found that introducing counter-swirl shifted the surge line toward lower flow rates. In separate investigations, Tokieda et al.^30^ and Tamaki et al.^15^ installed inlet fins upstream of the impeller leading edge, effectively reducing surge flow rates and broadening the operating range.

Further research by Gancedo et al.^31,32^, Ma and Kim^33^, Sivagnanasundaram et al.^34^, and Ruzicka^35^ examined the use of inlet bleeds and cavities for similar purposes. According to Gancedo et al.^32^, integrating bleed slots in the inducer region extended the compressor operating range without compromising performance, while open bleed slots helped suppress instabilities at low mass flow rates. Sivagnanasundaram et al.^34^ reported that increasing slot width expanded the map width by about 17%, albeit with a reduction in efficiency due to frictional and mixing losses. Broatch et al.^36^ analyzed how the inlet elbow radius affects backflow noise and compressor efficiency, revealing that a tighter radius increases inlet noise by distorting the incoming flow and amplifying backflow oscillations near surge. Similarly, Cravero et al.^37^ demonstrated that a ported shroud can enhance the surge margin by up to 11% at design speed and improve the pressure ratio near the surge boundary by approximately 7%.

In previous studies^15,30^ inlet fins were primarily analyzed using steady-state CFD simulations, with fins typically positioned before the diameter reduction, with the inner diameter aligned to the impeller shroud. These works largely neglected the transient behaviour and circumferential non-uniformity of inlet recirculation near the impeller inlet. In contrast, the present study addresses this gap by conducting a detailed transient CFD investigation of inlet fin configurations. The fins are positioned closer to the impeller inlet, within the contraction zone, allowing for an earlier and more realistic interaction with the evolving flow field. This approach provides a more comprehensive understanding of the unsteady mechanisms governing inlet recirculation and preswirl control, advancing beyond the steady-state assumptions of prior research.

### Goals and objectives

Inlet recirculation has been widely observed in various turbomachinery applications, yet it is not fully understood. A deeper understanding of this phenomenon is essential for optimizing compressor performance, particularly for extending the stable operating range and suppressing global instability. To address this challenge, inlet fins serve as a promising passive flow control solution for mitigating IR and stabilizing compressor operation. Positioned upstream of the impeller, these fins disrupt the swirl and suppress reverse flow propagation without degrading compressor performance. Due to their geometric simplicity, ease of installation, and practical applicability, inlet fins were chosen as the primary method for experimental validation in this study. The main objectives of this research are to (1) develop, validate, and assess the numerical model of the compressor, including discretization error evaluation; (2) investigate the influence of inlet fins on inlet recirculation characteristics and their impact on overall compressor performance and operating range.

## Methods

The investigations in this study are performed for a BorgWarner turbocharger compressor. The study combines experimental investigations with numerical simulations for detailed analysis. The experimental tests provide valuable performance data under control conditions, serving as a benchmark for validating computational models. The numerical approach involves reconstructing the compressor geometry, generating a fine mesh to capture flow features accurately, and setting up the solver to ensure robust simulations. The geometric and performance data for the compressor under study are reported in our previous study^19^.

### Experimental tests

**Fig. 1:**
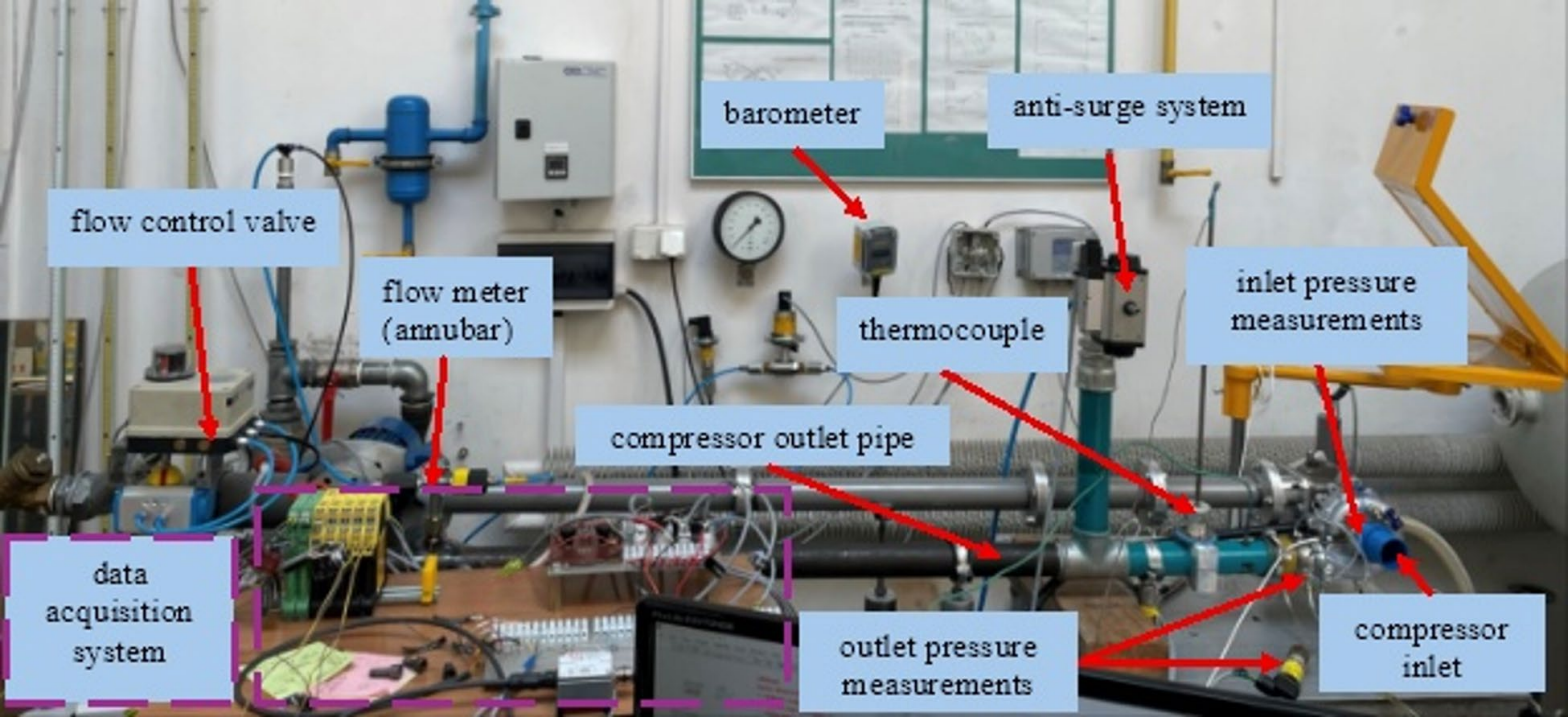
Photo of the experimental test stand with indication of the key measurement equipment.

The test stand located in the laboratory of the Institute of Turbomachinery, Lodz University of Technology, was used to investigate the performance of the BorgWarner turbocharger compressor under IR. The turbine, driving the compressor via a connecting shaft, is powered by air leaving the compressed air tanks, supplied by the screw compressor (GD-VSB11-3G 30/10) with the highest-pressure output of 1.0 MPa. The turbocharger compressor draws in ambient air through the compressor inlet, preceded by a short inlet pipe, compresses it, and discharges high-pressure air through the compressor outlet. To prevent compressor surge, an anti-surge system is installed in the outlet section of the compressor. The general view of the test stand with key elements of the installation is shown in Fig. 1. The ambient pressure during the experiments is measured by the Aplisens barometer (APC-2000 GN PCV). At the compressor outlet, the static pressure is measured by the Aplisens (PC-28) gauge pressure transducer with a range of 250 kPa. The ambient temperature is measured by a thermometer (NH506RA). The outlet static temperature is measured by the K-type thermocouple. The flow rate is measured by a mass flowmeter (Annubar GCR-15). The following equations were used to calculate the total-to-total pressure ratio and total-to-total isentropic efficiency of the centrifugal compressor.

1 $$\:{\pi\:}_{tt}=\frac{{p}_{t1}}{{p}_{t2}}$$

2 $$\:{\eta\:}_{is-tt}=\frac{{{\pi\:}_{tt}}^{\raisebox{1ex}{$\gamma\:-1$}\!\left/\:\!\raisebox{-1ex}{$\gamma\:$}\right.}-1}{\left(\frac{{T}_{t2}}{{T}_{t1}}\right)-1}$$

where $\:{p}_{t1}$ and $\:{p}_{t2}$ represent the total pressures at the compressor inlet (inlet pipe) and outlet (volute outlet), respectively; $\:\gamma\:$ is the ratio of specific heats at constant pressure and volume ($\:\gamma\:=\frac{{C}_{p}}{{C}_{v}}$ ); $\:{T}_{t1}$ and $\:{T}_{t2}$ denote the total temperatures at the compressor inlet (inlet pipe) and outlet (volute outlet), respectively.

The experiments were conducted at lower speed lines due to limitations in the available compressed air supply. The maximum pressure during the test was 800 kPa. Its output flow rate was insufficient to support the continuous operation of the turbocharger. Therefore, the compressed air system was equipped with two high-capacity storage (4 m^3^ each) tanks that acted as compressed air reservoirs during the experiment. This configuration allowed reliable testing within a controlled lower-speed operating range of the turbocharger.

Each measurement followed a standardized procedure to ensure repeatability. After charging the air tanks, the turbocharger was brought to the desired operating point by gradually opening the turbine control valve and allowing the system to stabilize. Flow parameters and compressor speed were monitored via the LabVIEW-based acquisition interface, with stabilization typically requiring 2–3 min. The acquisition system then recorded transducer signals for 5 s, after which the turbocharger was stopped. Before the next test point, the air tanks were recharged to the required pressure. This procedure ensured consistent data quality under controlled low-speed conditions. The raw data were subsequently processed in Excel to determine the relevant performance parameters. Three inlet configurations were examined experimentally to assess how inlet geometry influences centrifugal compressor performance. The baseline case used a smooth inlet, while the two modified cases incorporated medium-sized fins (F) and higher fins (H), respectively, both aimed at extending the compressor operating range. All inlet pipes, with and without fins, were 3D-printed and installed at the compressor inlet.

An uncertainty analysis was performed for the key performance parameters following the ISO GUM methodology, accounting in principle for both Type A and Type B uncertainties^38^. Because multiple repetitions were not available, only Type B uncertainties, arising from non-statistical sources such as calibration drift, manufacturer specifications, measurement resolution, model assumptions, and reference data, were evaluated. Standard uncertainties were assigned to each input variable based on its known error sources, and the combined uncertainties were obtained using root-sum-square propagation. For pressure ratio and isentropic efficiency, all relevant input quantities (pressures, temperatures, mass flow rate, and gas properties) were included. The pressure ratio showed a Type B standard uncertainty of ± 0.0027 to ± 0.0030, corresponding to a maximum relative uncertainty of 0.25% at the lowest mass flow rate. The isentropic efficiency uncertainty ranged from ± 0.004 to ± 0.014, with a maximum relative uncertainty of 2.6% at the highest mass flow rate, largely due to its sensitivity to temperature measurements. The mass flow rate exhibited a Type B uncertainty of ± 0.0004–0.0010 kg/s, with a maximum relative uncertainty of 5.1% at the lowest flow rate.

### Numerical methodology

The numerical approach used to analyze IR is outlined in the following subsections. The flow under investigation is unsteady, three-dimensional, compressible, and turbulent. Therefore, an unsteady RANS framework was adopted to capture the physics of the flow phenomena within available computational resources. All simulations were carried out using the ANSYS CFX solver, a widely established tool in turbomachinery applications. It was used for several research studies of the unsteady flow phenomena in compressors^34,39–41^.

### Computational domain

**Fig. 2:**
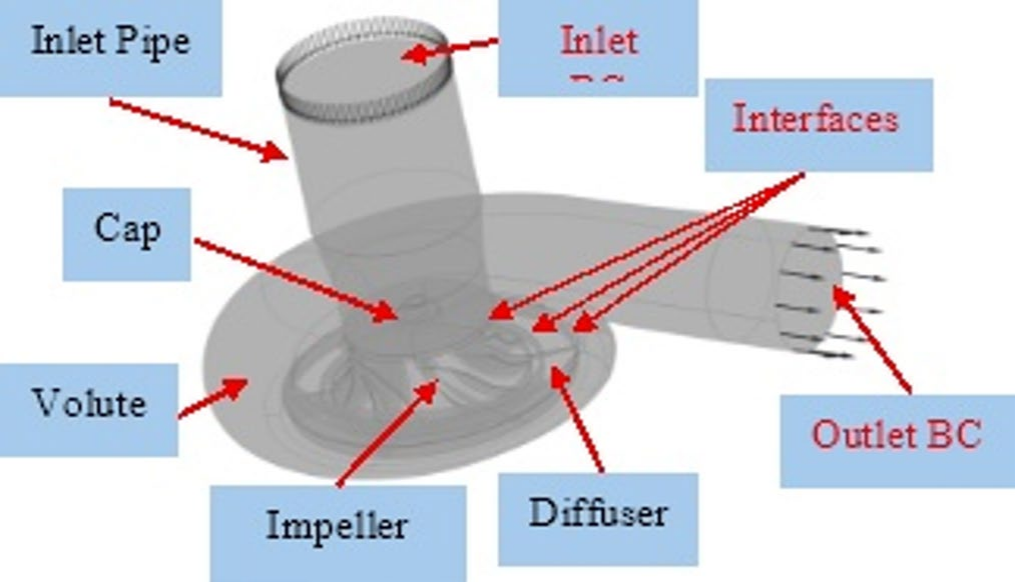
The turbocharger compressor computational domain.

The investigated numerical model reproduces the geometry of the main elements of the experimental test stand. Figure 2 illustrates the compressor computational domain, which includes the following components: (1) an inlet pipe; (2) an impeller; (3) a vaneless diffuser; and (4) a volute. The geometries of the impeller and volute of the turbocharger compressor were reconstructed using 3D laser scanning^42,43^.

### Mesh generation

**Fig. 3:**
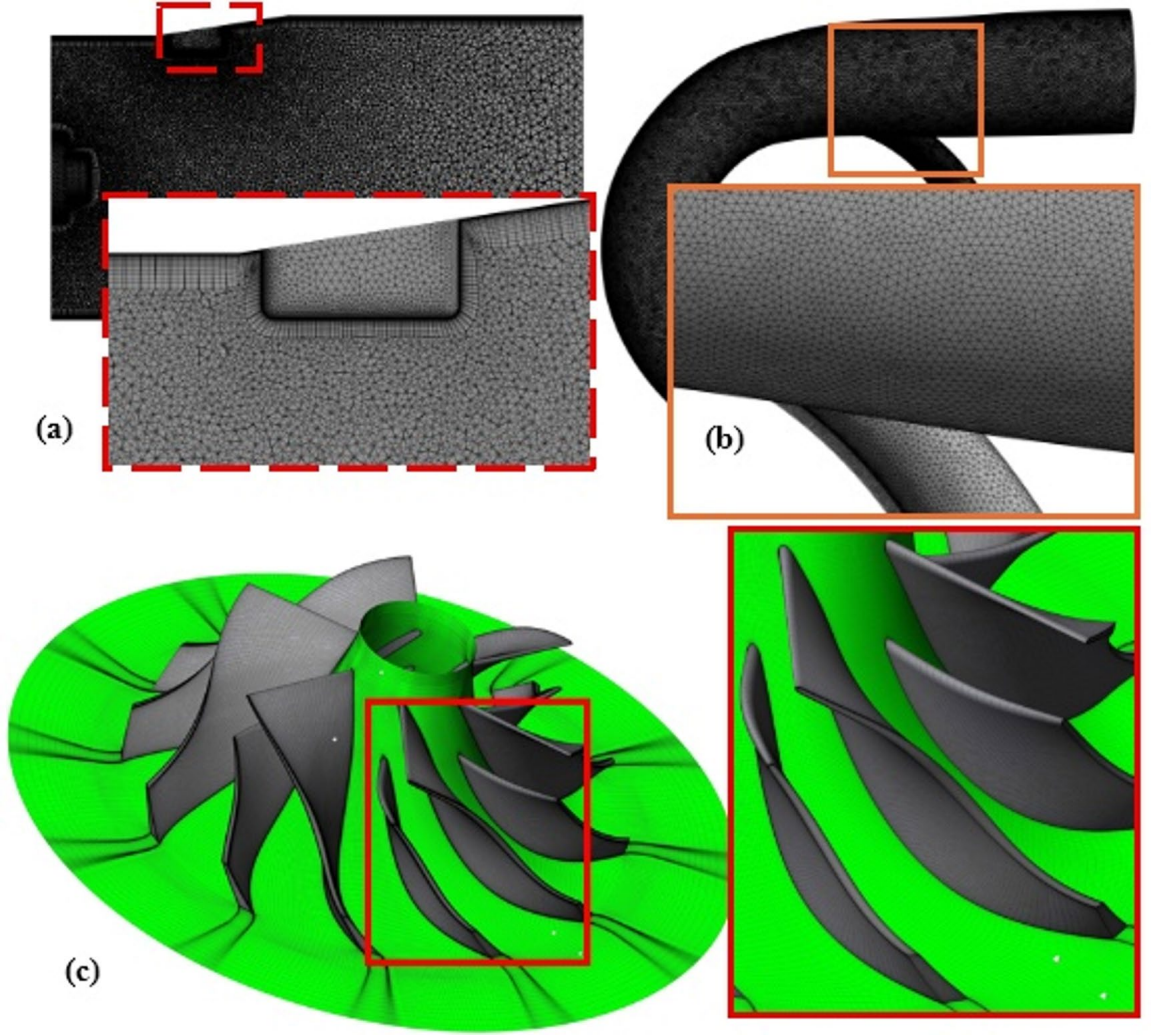
Meshes of main components: (**a**) inlet channel meridional cross-section with a zoomed view of a fin, (**b**) front view of the volute mesh with magnification of the volute region, (**c**) impeller mesh with magnifications of the blade regions (blades are presented in grey, and the hub surface is presented in green).

ANSYS CFX employs a finite-volume approach, in which the CAD-defined fluid domains are divided into control volumes where the discretized conservation equations of mass, momentum, and energy are solved^44^. Accurate mesh resolution is essential for capturing the complex flow structures within the stationary and rotating components of the compressor. Two meshing tools were used: ANSYS TurboGrid for the impeller and vaneless diffuser, producing a high-quality structured mesh based on combined H- and O-grid topologies (O-grids around the blades and H-grids elsewhere). The inlet pipe and volute were meshed in ANSYS Meshing using unstructured tetrahedral elements with 25 prismatic wall layers. The wall-resolved mesh achieved y⁺ < 1 (non-dimensional wall distance) throughout most regions, except for slightly higher values at the impeller trailing edges, which did not affect solution accuracy. This ensured adequate resolution of the viscous sublayer, which is essential for predicting flow separation. Figure 3 illustrates meshes of the inlet pipe, volute, and the impeller with magnification of the leading edges of both the main and splitter blades. Mesh sizes were selected based on a mesh-independence study^19^. The final meshes of the inlet pipe contained 5.56 and 9.12 million elements for the configurations without and with fins, respectively, while the impeller and volute meshes contained 10.68 and 9.6 million elements, respectively.

### Solver setup

Due to the complexity of the flow and computational constraints, the RANS approach with the SST turbulence model was employed, a widely used method for simulating unsteady compressor phenomena^45–47^. This methodology has also been effectively applied in prior studies at the Institute of Turbomachinery^19,48,49^. Numerical simulations were conducted in ANSYS CFX 2022 R2 under both steady and transient conditions. Air was modeled as an ideal gas within the given pressure and temperature ranges. The inlet and outlet domains (inlet pipe, vaneless diffuser, and volute) remained stationary, while the impeller domain rotated at a case-specific rotational speed *n*.

Following prior studies^46,47^ and earlier research at the Institute of Turbomachinery^19,48,50^, total pressure and mass flow rate were applied as boundary conditions at the inlet pipe and volute outlet, respectively. This configuration provides accurate flow predictions near the nominal and reduced flow rates but is less effective for choked conditions. The inlet total pressure and temperature were set to 100 kPa and 298 K, consistent with the experimental setup. The inlet turbulence intensity was 5%, with an eddy-to-molecular viscosity ratio of 10. The outlet mass flow rate $\dot{m}$ varied according to each operating point. All walls were modeled as no-slip, smooth, and adiabatic.

Different interface models were applied to account for the change in reference frames between stationary and rotating domains. For steady-state simulations, frozen-rotor interfaces were used between the inlet pipe and impeller, and between the impeller and vaneless diffuser, allowing recirculation effects to be preserved. For transient simulations, transient rotor–stator interfaces were employed at both locations. Because the meshes between the vaneless diffuser and volute were non-conformal, a General Grid Interface (GGI) was used to enable data exchange between these domains^51^.

In this study, steady-state solutions primarily provided initial conditions for transient simulations. The transient computations were iterated until the conservation equations converged within each timestep. Based on prior research and preliminary assessment, the initial timestep corresponded to a 3° impeller rotation, later refined to 1° once global parameters stabilized. For the design speed line of 370 m/s (*n* = 136,200 rpm), the corresponding timesteps were 3.67.10⁻⁶ s and 1.22.10⁻⁶ s, respectively. The same approach was applied to other rotational speeds.

For both steady-state and transient simulations, the high-resolution advection scheme was used to discretize the conservation equations. This method applies a second-order upwind scheme except in regions with steep gradients, where the discretization order is locally reduced^51^. The turbulence model equations were also solved using a second-order scheme. In transient simulations, time integration employed the second-order backward Euler method. Convergence criteria were defined with Root Mean Square (RMS) residuals of 1e-5 for steady-state and 2e-5 for transient cases. During each transient timestep, up to 10 internal iterations were performed to meet the specified convergence targets.

In CFD simulations, achieving a high accuracy of the solution is essential. One of the possible sources of errors is inadequate mesh refinement, making a discretization uncertainty assessment or mesh independence study critically important. A common method to validate mesh reliability is the Richardson Extrapolation (RE) method of discretization uncertainty estimation described by Celik et al.^52^, which is nowadays a standard approach in the field of CFD. To establish a suitable grid size for simulating a centrifugal compressor, an evaluation of discretization-related uncertainty was performed using the aforementioned RE methodology. Steady-state simulations at the design point and a point on the surge line were performed. Three different grid sizes: coarse (*15.74 million elements*), medium (*25.78 million elements*), and fine (*40.57 million elements*) were used. The key parameters of the compressor performance: the total-to-total pressure ratio and the total-to-total isentropic efficiency, whose average values were used as evaluation criteria for assessing simulation convergence. The key results of this analysis were published in our previous study^19^. At the design point, both pressure ratio and isentropic efficiency achieved satisfactory mesh convergence, with GCI values of 2% and 3.4%, respectively. Near the surge line, the corresponding GCI values were 1.7% for pressure ratio and 1.2% for efficiency. The medium- and fine-mesh solutions showed minimal differences; thus, the medium mesh was adopted for subsequent simulations. With this mesh, the pressure ratio and isentropic efficiency differed from the extrapolated values by only 2.0% and 3.8%, respectively.

## Results and discussion

**Fig. 4:**
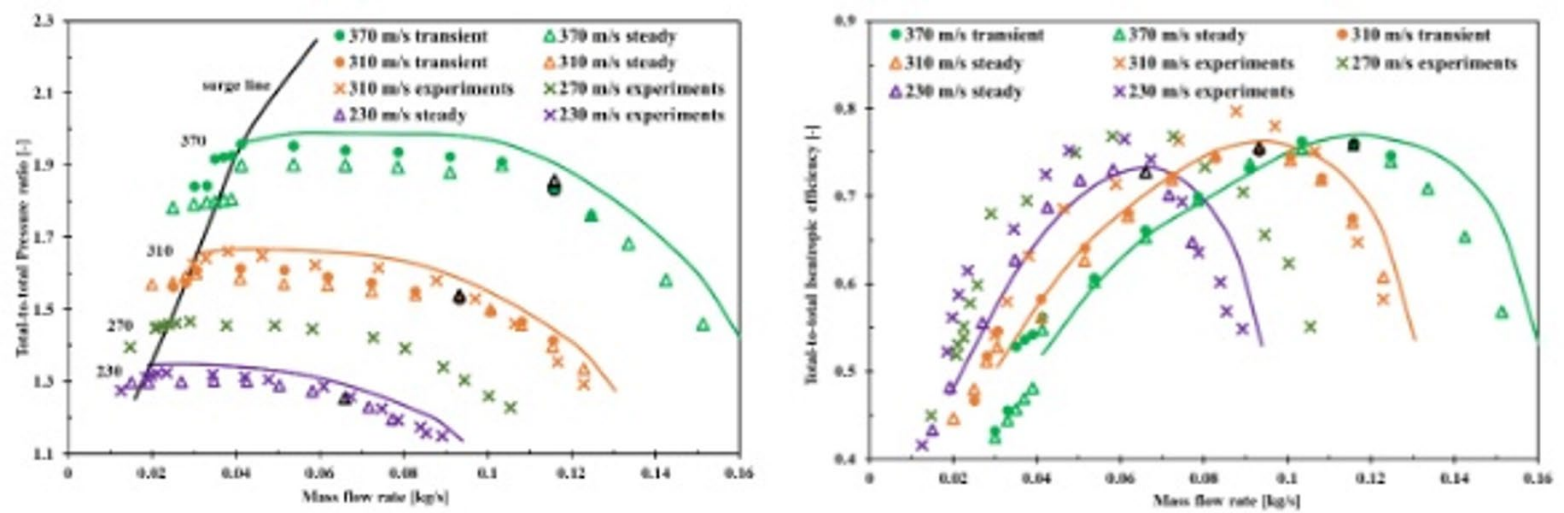
Total-to-total pressure ratio (left) and isentropic efficiency (right) comparison: simulation vs. manufacturer data and own experiment.

The study examined the inlet recirculation phenomenon in a turbocharger compressor and assessed the effects of inlet fins using both steady-state and transient CFD simulations across three rotational speeds: 370 m/s, 310 m/s, and 230 m/s. Due to limitations in the test stand, only the lower speed lines (310 m/s, 270 m/s, and 230 m/s) were investigated experimentally, while the nominal speed line (370 m/s) served for comparison of numerical simulation results with manufacturer data. Figure 4 presents numerical simulation results and manufacturer and experimental data for total-to-total pressure ratio and isentropic efficiency as functions of mass flow rate. Manufacturer data are shown as solid lines, experimental results as crosses, and numerical results as solid circles (transient) and hollow triangles (steady-state). Design points at 370 m/s and peak-efficiency points at 310 m/s and 230 m/s are marked with black symbols.

For the 370 m/s speed line, transient simulations consistently underpredict the manufacturer’s pressure ratio (Fig. 4 left), being 3.9% lower at the design point (0.116 kg/s) and 0.2% lower at the surge line (0.041 kg/s), with a maximum deviation of 4.6% (0.125 kg/s). Steady-state results show larger deviations, particularly near the choke limit, reaching 7.8%, while near the design point, the difference is 2.6%. For the 310 m/s speed line, the experimental data align well with the manufacturer’s curve but underpredict at higher flow rates, with a 6.8% deviation near the choke, 3.7% at the design point, and 1.8% at the surge line. Transient simulations follow the trend closely, showing a maximum deviation of 4.6% (0.083 kg/s), while steady-state results differ by up to 6.0% (0.072 kg/s). At the surge line, deviations are 2.8% (transient) and 3.4% (steady-state); near peak efficiency (0.093 kg/s), they are 3.7% and 3.1%, respectively. For the lowest speed line of 230 m/s, experimental results closely match the manufacturer’s curve with a maximum deviation of 4.6%, while steady-state simulations show up to 4.1% difference and a 3.6% drop at the surge line.

Figure 4 (right) shows clear discrepancies in total-to-total isentropic efficiency. Numerical simulations significantly underpredict efficiency at high flow rates but show smaller deviations at low flows. For the 370 m/s line, efficiency is 1.3% (transient) and 1.6% (steady-state) lower at the design point, while overpredicted by 8.3% and 5.5% at surge, respectively. The 310 m/s line follows a similar trend: underprediction above 0.0932 kg/s, minimal deviation at peak efficiency (1.3% transient, 1.0% steady-state), and overprediction near surge (4.4%, 8.1%). For 230 m/s line, steady-state results are 0.7% below manufacturer data at peak efficiency and 1.7% difference at surge. Experimental efficiencies for 310 m/s and 230 m/s lines generally match manufacturer trends but deviate across operating conditions. At high flow rates, experiments underpredict efficiency, closely matching simulations, while at medium and low flows, they often overpredict. For 310 m/s, deviations reach 12.8% near choke and 10.7% at low flows, reducing to 2.6% at peak efficiency and exceeding 6.7% at surge. For 230 m/s, experimental efficiencies are 1.2% and 15.6% higher at the peak and surge points, respectively.

Overall, a satisfactory correlation is observed among the experimental data, numerical simulations, and the manufacturer’s performance curves across both design and surge line conditions. Reported discrepancies are consistent with literature findings^17,53,54^. For example, a study by Cui et al.^53^ noted relative errors of 2.3–3.6% between experimental and CFD results near the choke and design points, while Lin et al.^17^ and Dewar et al.^54^ observed comparable deviations of 2–5%. Transient simulations demonstrate improved prediction of the pressure ratio at low mass flow rates, where steady-state models struggle to capture unsteady flow phenomena. Deviations at low-flow and off-design conditions are primarily attributed to modeling constraints such as mesh resolution, boundary condition settings, domain configuration, as well as different locations of the measurement points in the manufacturer’s test stand. Minor geometric deviations due to manufacturing tolerances or thermal loading may further contribute. Despite these limitations, the numerical predictions show satisfactory accuracy, with performance trends consistent with both experimental and reference data.

Inlet recirculation intensifies near the surge line and disrupts flow uniformity, leading to degraded compressor performance^4,17^. Literature findings^15,30^, suggest that mitigating this phenomenon can extend the stable operating range of centrifugal compressors. Based on these insights, the introduction of inlet fins ahead of the impeller was investigated as a potential flow-control strategy. Subsequent sections compare the compressor configurations with and without inlet fins to evaluate their impact on overall performance and surge margin.

### Selection of the inlet fin configuration

**Fig. 5:**
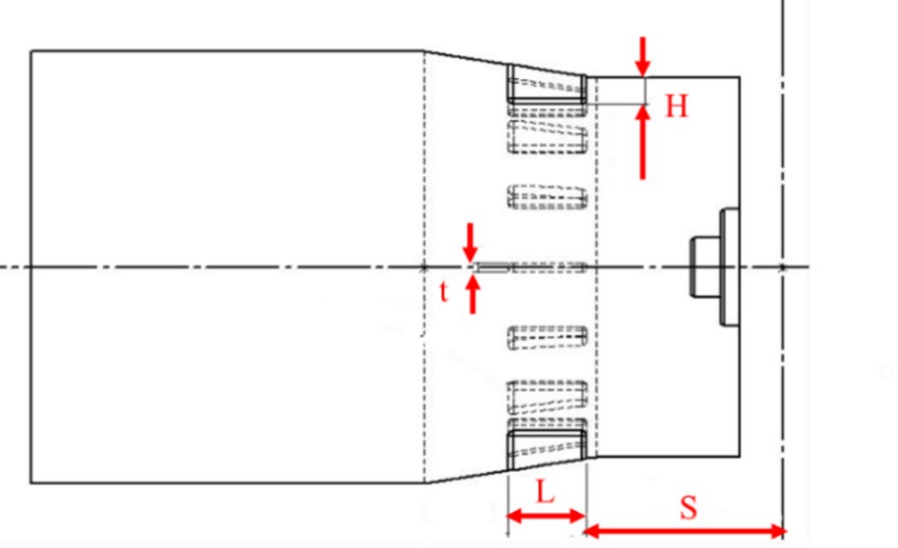
Sketch of the inlet channel with fins.

The inlet fin configuration was selected based on steady-state simulations performed at the surge-line operating point for the design speed of 370 m/s. Various fin geometries were assessed; however, following literature insights^15,30^, and preliminary tests, constant values of fin length (L = 8 mm), thickness (t = 1 mm), and number (*N* = 16) were maintained. This configuration effectively reduced rotational flow in the recirculation zone while minimizing blockage and ensuring sufficient structural strength. Consequently, the study focused on varying fin height (H) and axial distance from the impeller leading edge (S) to evaluate their influence on inlet flow behavior. Figure 5 illustrates the sketch of the inlet channel featuring inlet fins. A vertical black dash-dot line on the right side marks the location of the impeller leading edge. Table 1 provides specific information about the various inlet fin arrangements. Three fin positions, upstream of, within, and downstream of the inlet pipe contraction, were analyzed, each with different fin heights. These configurations enabled a systematic assessment of how fin geometry affects inlet recirculation and overall compressor flow characteristics.

**Table 1:** Inlet fins configuration details.

Cases	A	B	C	D	E	F	G	H	I	J
**Fins height H [mm]**	2.0	3.0	4.0	1.5	2.0	3.0	4.0	5.5	4.0	7.0
**Position from LE S [mm]**	38	38	38	20	20	20	20	20	11	11

**Fig. 6:**
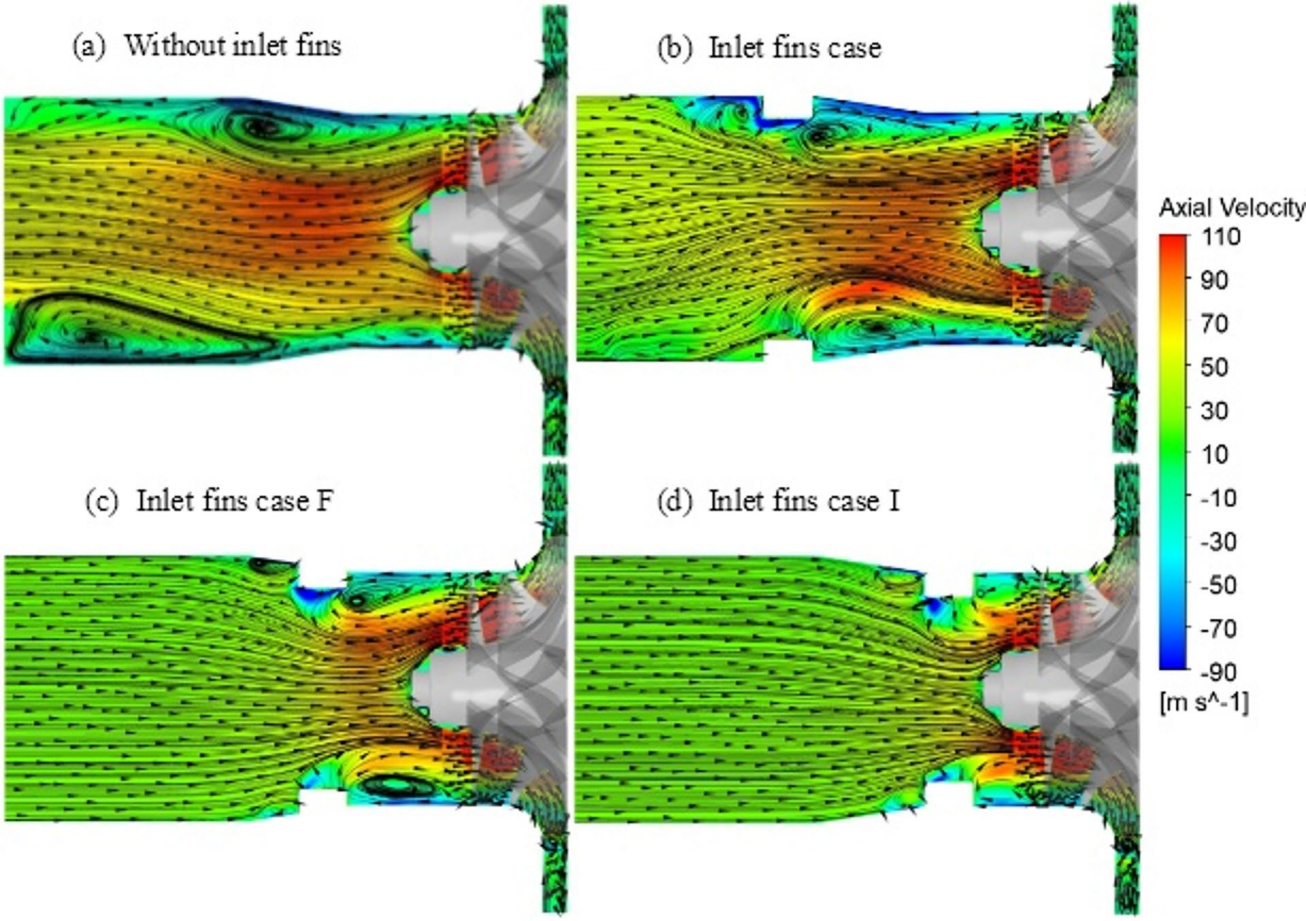
Flow patterns in the meridional section at the impeller inlet for selected inlet fin configurations for the point on the surge line for the 370 m/s speed line (steady-state simulations; streamlines with a meridional velocity component in the background).

Figure 6 presents meridional flow patterns at the compressor inlet for configurations with and without inlet fins, illustrating the impact on flow uniformity and recirculation. Configurations C, F, and I were identified as optimal for their respective axial positions (S distances), while other simulated fins configurations showed inferior performance. In the baseline case without fins, inlet recirculation extended significantly upstream. When fins were placed farther from the impeller (Case C), reversed flow was mitigated, but inlet non-uniformity persisted. Locating fins within the contraction zone (Case F) produced the most uniform flow, effectively suppressing upstream recirculation, while fins placed closer to the impeller (Case I) yielded similar improvements. These results confirm that inlet fins prevent recirculation from propagating upstream, though they do not substantially alter the near-impeller flow and may introduce minor local disturbances. At the design point, performance differences between the finned and baseline configurations were negligible: the pressure ratio increased marginally from 1.763 to 1.764 (0.06%), and isentropic efficiency decreased slightly from 0.7237 to 0.7236 (0.01%). The findings suggest that incorporating fins does not negatively impact compressor efficiency at the design operating condition.

**Fig. 7:**
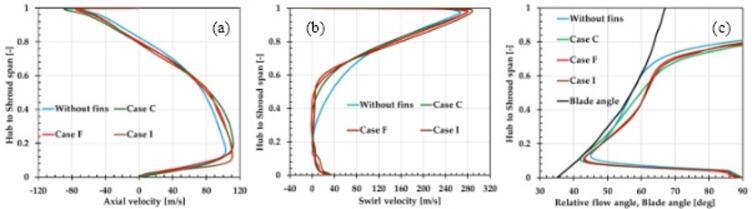
Axial (**a**), circumferential (**b**) velocity components, and relative flow angle and blade angle (**c**) profiles along the span at the control plane at the impeller domain inlet for cases with and without inlet fins for the point on the surge line for the 370 m/s speed line (steady-state simulations; parameters averaged circumferentially).

Figure 7 (a), (b) compares circumferentially averaged axial and swirl velocity profiles for selected fin configurations (C, F, and I) with the baseline case without fins, evaluated at the control plane between the inlet pipe and impeller. The introduction of fins produced notable changes in swirl velocity, with significant suppression of upstream swirl that limits momentum exchange between recirculating and core flows. Consequently, the circumferential velocity component decreased below 0.7 of the span, and fins positioned closer to the impeller shifted the zero-swirl region toward higher spans. However, near the shroud, the swirl velocity increased relative to the no-fin case. In terms of axial velocity, a moderate increase was observed from the hub to mid-span, while the reverse flow near the shroud intensified. Relative flow angle and blade angle profiles at the impeller leading edge (Fig. 7 (c)) indicate that inlet fins increase the relative flow angle, particularly from mid-span to shroud, resulting in higher incidence due to reduced preswirl. In contrast, the no-fin configuration exhibited a lower incidence owing to stronger inlet recirculation. Among the finned setups, cases F and I showed nearly identical relative flow angle distributions and the highest incidence levels.

The analysis revealed that inlet fins significantly influence the impeller inlet flow. Proper fin height effectively suppresses upstream swirl, while axial placement critically determines the extent of flow control and recirculation mitigation, aligning with findings of Tamaki et al.^15^. Fins positioned too far upstream failed to prevent reverse flow, whereas those placed too close to the impeller induced harmful disturbances and fatigue risks. Positioning fins within the contraction zone offered an optimal balance, sufficiently distant to avoid impeller interference yet effective in damping swirl and promoting uniform inflow. Consequently, configuration F was selected for transient and experimental analysis, as it minimized inlet recirculation and improved flow uniformity. Unlike prior studies^15,30^, which favored upstream fin placement, this work positioned fins closer to the impeller, achieving superior swirl suppression, albeit with greater integration challenges in existing turbocharger designs.

**Fig. 8:**
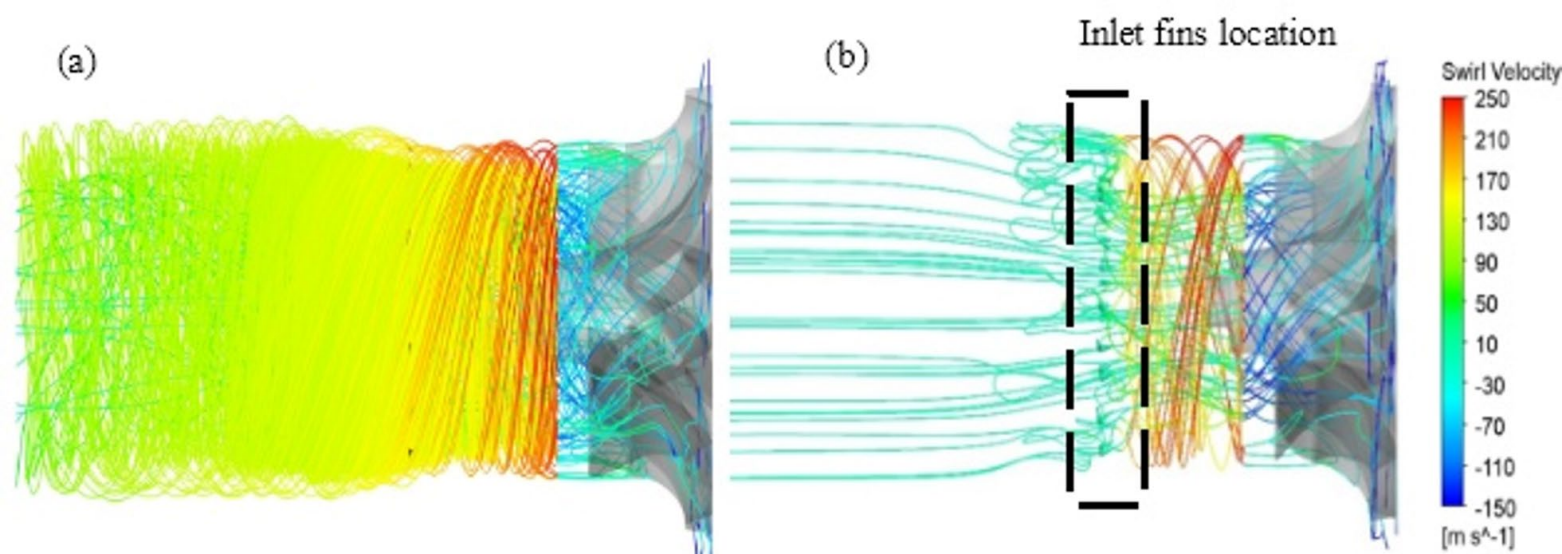
Streamlines for the time-averaged flow for the point on the surge line (**a**) without and (**b**) with inlet fins (case F) (streamlines colors correspond to the circumferential velocity component).

**Fig. 9:**
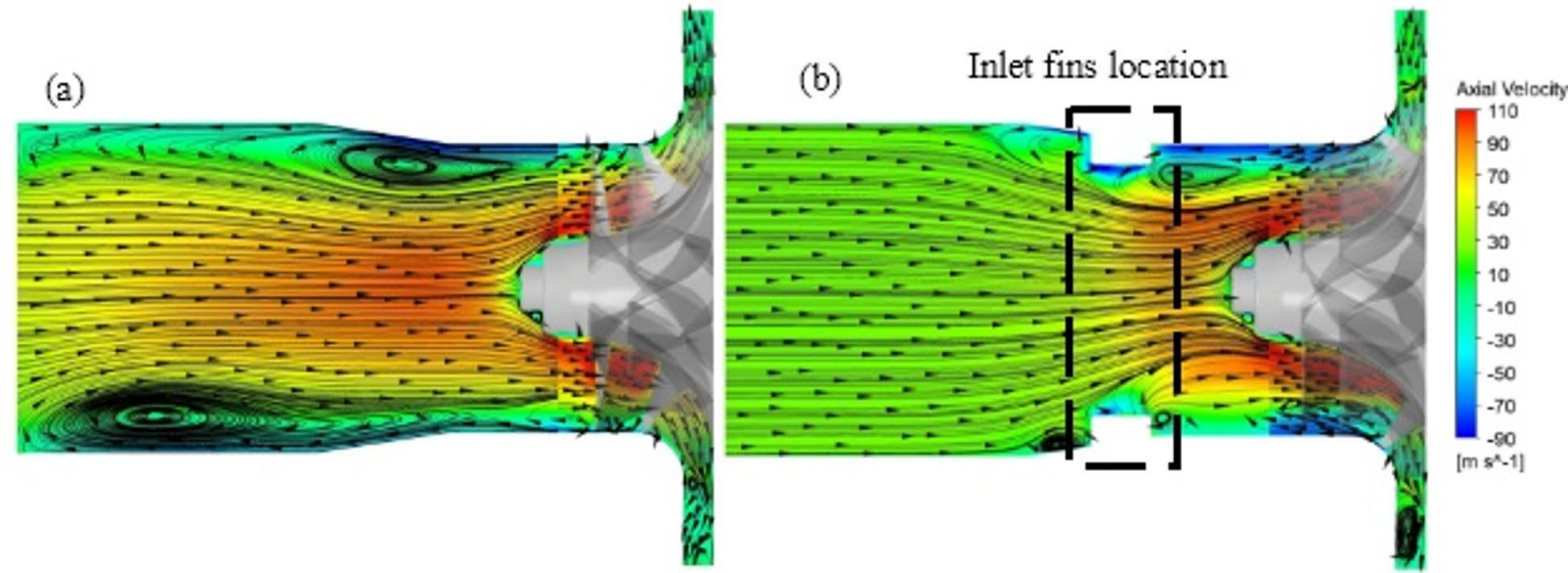
Time-average flow patterns in the meridional section at the impeller inlet for the point on the surge line for the 370 m/s speed line: (a) without and (b) with inlet fins (Case F).

**Fig. 10:**
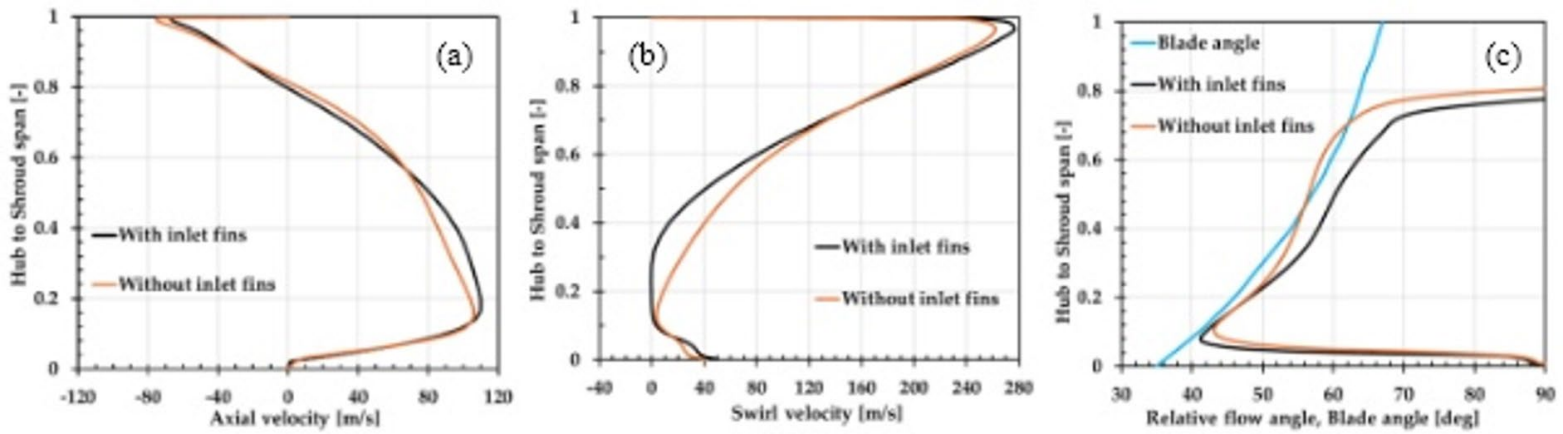
Axial (**a**), circumferential (**b**) velocity components, and relative flow angle and blade angle (**c**) profiles with inlet fins (Case F) and without inlet fins for the point on the surge line for 370 m/s speed line (transient simulations; parameters were first time-averaged and then circumferentially averaged).

Transient simulations were performed for the compressor equipped with fins in configuration F at the surge line operating point for the design speed (370 m/s) and compared against the baseline case without fins. The results validated the steady-state predictions regarding fin performance. As shown in Figs. 8 and 9, the absence of fins allows reverse flow with a strong swirl component to propagate upstream toward the compressor inlet, whereas the inclusion of fins significantly alters the flow structure. The fins effectively disrupt the reverse flow, dissipating the swirling motion and confining recirculation to a shorter upstream region. This reduction in recirculation limits angular momentum transfer to the core flow, thereby diminishing preswirl and promoting a more uniform velocity distribution at the impeller inlet. Figure 10 (a), (b) further illustrates that the fins notably reduce swirl velocity from the hub to mid-span under transient conditions, although the suppression is less pronounced than in steady-state simulations (Fig. 7). The axial velocity component shows minimal variation; nonetheless, the fin-equipped configuration consistently demonstrates improved swirl suppression in both steady and unsteady regimes.

Figure 10 (c) compares the relative flow angle and blade angle profiles at the impeller leading edge for the surge condition, with and without inlet fins. The results indicate that the inlet fins (black line) produce a higher relative flow angle, particularly from mid-span to the shroud, compared to the configuration without fins (orange line). This increased deviation between the flow and blade angles reflects a higher incidence angle, which can reduce efficiency under off-design conditions. Conversely, in the absence of fins, the relative flow angle aligns more closely with the blade angle due to the preswirl generated by inlet recirculation near surge, thereby lowering the impeller incidence.

### Improvement of the operating range by inlet fins

The influence of inlet fins on the compressor operating range was examined through a combination of experiments and numerical simulations. Experimental investigations were conducted only for 230 m/s and 270 m/s speed lines due to limitations in the experimental setup, specifically, the insufficient supply of compressed air. Although preliminary measurements were performed at the 310 m/s speed line for the no-finned configuration, reliable data near the surge limit could not be obtained. This was mainly due to the prolonged stabilization time required for temperature measurements at the compressor outlet and the need for gradual adjustment of operating conditions to avoid surge onset. The installation of inlet fins improved inlet flow uniformity and reduced recirculation; however, this enhancement did not sufficiently reduce stabilization time or eliminate the unsteadiness associated with near-surge operation. Consequently, the available compressed air was depleted before stable measurements could be achieved at this speed. Therefore, numerical simulations were employed to complement the experimental data at higher speeds (310 m/s and 370 m/s) for the F-configuration inlet fins, providing additional insight into recirculation control and compressor performance under near-surge conditions.

**Fig. 11:**
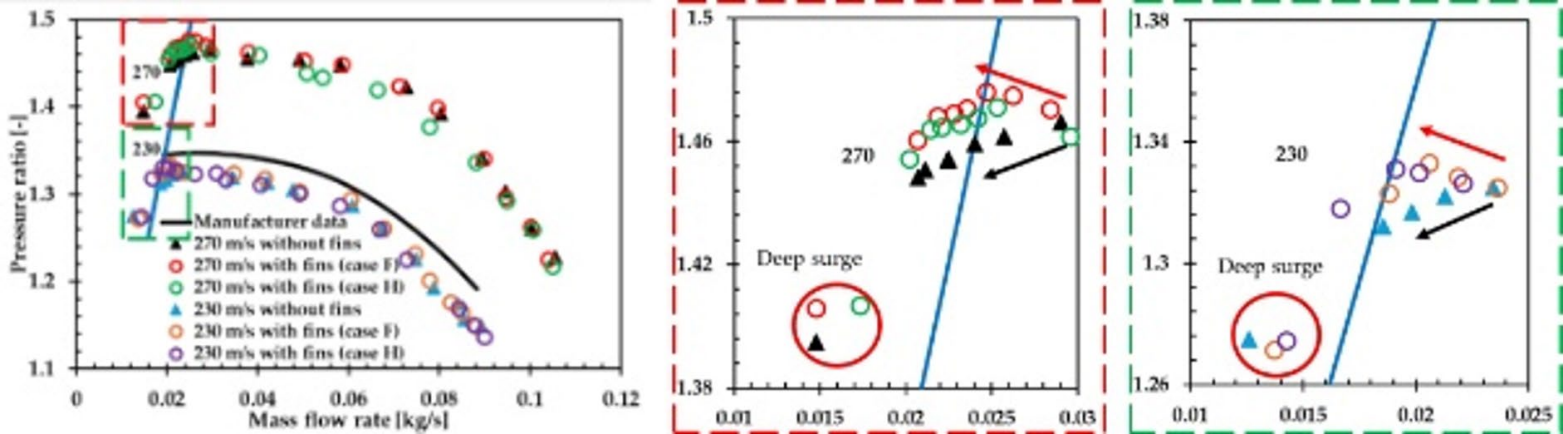
Total-to-total pressure ratio determined experimentally for inlet configurations with and without fins, with the zoomed view of 270 m/s (red dashed box) and 230 m/s (green dashed box) speed lines results.

Figure 11 compares the experimental results for finned and non-finned configurations in terms of pressure ratio as a function of mass flow rate. The solid black line denotes the manufacturer’s baseline performance curve, while the symbols represent measured data for three cases: without fins (black and light blue triangles), with fins (case F) (red and orange circles), and with higher fins (case H) (green and violet circles). The zoomed-in regions at lower mass flow rates illustrate the performance shift between the configurations, with arrows indicating the direction of change.

For both speed lines (230 m/s and 270 m/s), experimental results show that finned (case F) and no-finned configurations yield nearly identical pressure ratios at medium to high mass flow rates. This indicates that inlet fins have minimal influence on compressor aerodynamics under stable, high-flow conditions without flow instabilities or inlet recirculation. At flow rates below peak efficiency, differences remain negligible due to the limited effect of fins on weak or moderate recirculation. The configuration with higher fins (case H) shows slightly lower pressure ratios, likely due to increased inlet blockage and disturbance caused by the fins. In contrast, at low mass flow rates near the surge line, fins noticeably improve compressor performance. Both finned cases exhibit higher pressure ratios than the no-finned configuration, maintaining increases of 1.5% (270 m/s) and 1.1% (230 m/s) at the surge line. Beyond this point, the pressure ratios for finned cases decline but remain above those without fins. The high-fin configuration (case H) again shows marginally lower ratios than case F. Compressor operation was sustained down to approximately 0.020 kg/s (270 m/s) and 0.017 kg/s (230 m/s) before the anti-surge system engaged, with only minor differences observed between finned and no-finned setups in the onset of deep surge.

**Fig. 12:**
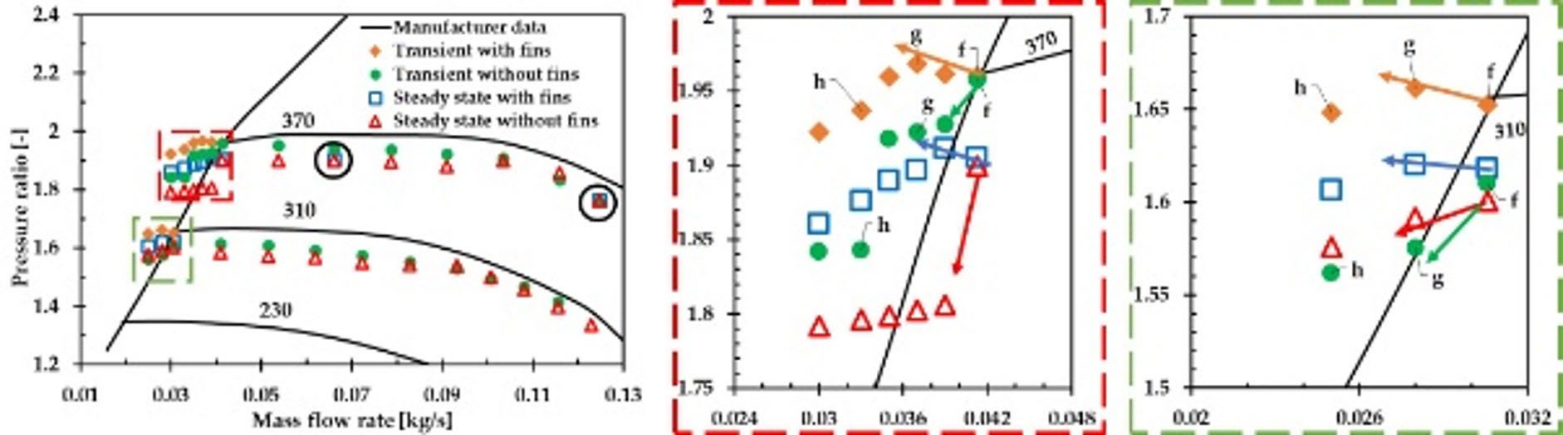
Total-to-total pressure ratio determined numerically for inlet configurations with and without fins, with the zoomed view of 370 m/s (red dashed box) and 310 m/s (green dashed box) speed lines results.

Numerical simulations conducted at higher speed lines (310 m/s and 370 m/s) also confirm the positive influence of inlet fins on the compressor pressure ratio. As shown in Fig. 12, pressure ratio variations for configurations with and without fins demonstrate that fins do not adversely affect compressor performance under stable operating conditions. Only two points (highlighted by black circles) were simulated to validate this observation, as special interest is in the range of the flow rates beyond the surge line. In these plots, the solid black line denotes the baseline performance curve provided by the manufacturer, while filled and open symbols represent transient and steady-state results, respectively, for both finned and no-finned cases. Enlarged views with arrows illustrate the performance shifts between configurations.

Although the numerical setup did not permit a direct surge analysis, the pressure ratio trends reveal unfavorable flow behavior at low mass flow rates. In the no-finned configuration, both transient and steady-state simulations show a pronounced reduction in pressure ratio beyond the surge line. For the 370 m/s speed line, steady-state results indicate a 5.2% drop in pressure ratio immediately past the surge line, signifying degraded flow conditions. Transient simulations show a more gradual decline, initially by 1.6%, then progressively down to 6.2% at 0.033 kg/s. A similar pattern is observed for the 310 m/s line, where the transient pressure ratio decreases by 2.2% beyond the surge line. These findings suggest that, in numerical analyses, compressors without inlet fins experience a deterioration in pressure ratio just past the surge line, even though the configuration prevents direct surge prediction.

As discussed earlier, inlet fins mitigate inlet recirculation by preventing reversed flow from propagating upstream in the inlet duct. Consequently, at the same mass flow rates, the compressor receives a more uniform and directed inflow, leading to improved impeller performance and a higher pressure ratio. At the 370 m/s speed line, simulations at the manufacturer-defined surge point show a slight pressure ratio increase with fins, about 0.1% in transient and 0.3% in steady-state conditions. Beyond the surge line, this effect becomes more pronounced: while the no-finned configuration exhibits a decline in pressure ratio, finned cases show increases of 1.8% and 2.4% (point g) in steady-state and transient simulations, respectively. With further decreases in mass flow rate, gradual declines appear in both simulation types. A similar pattern occurs at 310 m/s. At the surge point, fins improve the pressure ratio by 1.1% in steady-state and 2.6% in transient simulations. Beyond this point, the improvements reach 1.8% and 5.4%, respectively, confirming that inlet fins enhance flow stability and expand the stable operating range across both speed lines. These numerical findings align well with the experimental results. The inclusion of inlet fins also shifts the surge flow rate, defined by Sivagnanasundaram et al.^26^ as the mass flow rate at which total pressure begins to drop, marking the onset of surge instability. The improvement is quantified by the surge enhancement metric $\:{\Delta\:}{\dot{m}}_{\mathrm{s}}$, defined as:

3 $$\:{\Delta\:}{\dot{m}}_{\mathrm{s}}=\left(1-\frac{{\dot{m}}_{\mathrm{s}\mathrm{u}\mathrm{r}\mathrm{g}\mathrm{e}\_\mathrm{f}\mathrm{i}\mathrm{n}\mathrm{s}}}{{\dot{m}}_{\mathrm{s}\mathrm{u}\mathrm{r}\mathrm{g}\mathrm{e}\_\mathrm{w}\mathrm{i}\mathrm{t}\mathrm{h}\mathrm{o}\mathrm{u}\mathrm{t}\:\mathrm{f}\mathrm{i}\mathrm{n}\mathrm{s}}}\right)\mathrm{*}100\mathrm{\%}$$

where $\:{\dot{m}}_{\mathrm{s}\mathrm{u}\mathrm{r}\mathrm{g}\mathrm{e}\_\mathrm{f}\mathrm{i}\mathrm{n}\mathrm{s}}$ is the surge mass flow rate with the inlet fins (point g in Fig. 12) and $\:{\dot{m}}_{\mathrm{s}\mathrm{u}\mathrm{r}\mathrm{g}\mathrm{e}\_\mathrm{w}\mathrm{i}\mathrm{t}\mathrm{h}\mathrm{o}\mathrm{u}\mathrm{t}\:\mathrm{f}\mathrm{i}\mathrm{n}\mathrm{s}}$ is the surge mass flow rate without fins (point f Fig. 12). In transient simulations, $\:{\Delta\:}{\dot{m}}_{\mathrm{s}}$ reached 10.4% for 370 m/s and 8.4% for 310 m/s, indicating a substantial extension of the compressor stable operating range. In the experimental investigations, improvements of approximately 14.9% and 12.2% were observed for the 270 m/s and 230 m/s speed lines, respectively. These results are similar with the observations of Tamaki et al.^15^, who reported stability limit improvements of 4.6–11% with inlet fins across varying compressor speeds.

**Fig. 13:**
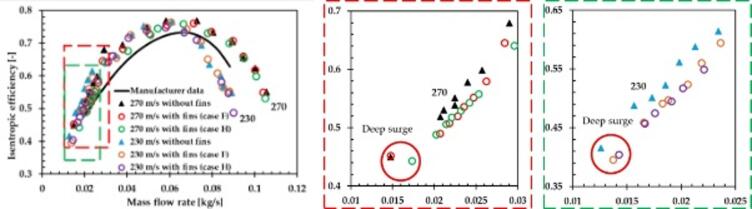
Total-to-total isentropic efficiency determined experimentally for inlet configurations with and without fins, with the zoomed view of 270 m/s (red dashed box) and 230 m/s (green dashed box) speed lines results.

**Fig. 14:**
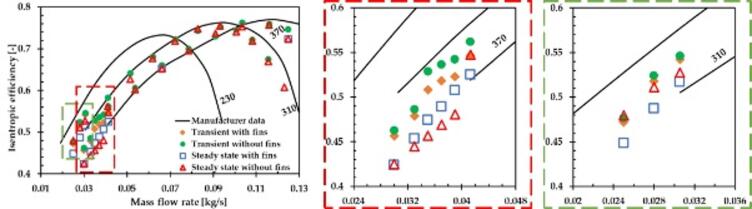
Total-to-total isentropic efficiency determined numerically for inlet configurations with and without fins, with the zoomed view of 370 m/s (red dashed box) and 310 m/s (green dashed box) speed lines results.

Figures 13 and 14 present the total-to-total isentropic efficiency obtained from experimental and numerical analyses, respectively. Since overlapping data obscure efficiency trends, separate zoomed-in views for each speed line are shown (red and green dashed boxes) to highlight the low-flow-rate region.

Experimentally, for both rotational speeds (270 m/s and 230 m/s tip speeds), the finned configurations (Cases F and H) show no efficiency deterioration relative to the no-finned case at high flow rates, i.e., above the efficiency peak. At lower flow rates, a slight decrease is observed, more pronounced for Case H. As the flow rate approaches surge conditions, efficiency declines across all configurations. For the 270 m/s speed line, near the manufacturer’s surge point (0.029 kg/s), efficiency drops by 5% and 5.7% for Cases F and H, respectively, compared with the no-finned configuration. This difference remains nearly constant at even lower flow rates. At 0.025 kg/s, where the pressure ratio improves, the efficiency of Case F is 1.5% lower, and that of Case H is 0.1% lower than the no-finned configuration. A similar pattern is observed at 230 m/s. At the stability limit (0.020 kg/s), efficiency reductions of 5.1% for Case F and 5.6% for Case H are recorded. For smaller flow rates, the differences diminish slightly and fall within the measurement uncertainty. The data points corresponding to the lowest flow rates at both speed lines represent deep surge conditions and were collected just before activation of the anti-surge system; therefore, they should not be considered representative.

In the numerical simulations, the influence of inlet fins on compressor efficiency follows a pattern similar to that observed for the pressure ratio. At high and moderate flow rates, fins have no significant effect on efficiency. However, as shown in Fig. 14, a noticeable decline in isentropic efficiency occurs beyond the surge line, consistent with the experimental findings. For the 370 m/s speed line, discontinuities in efficiency correspond to abrupt pressure ratio changes. In steady-state simulations, the no-finned configuration exhibits a 13.9% efficiency drop immediately beyond the surge line. Transient simulations show a more gradual trend, with smaller decreases down to 0.035 kg/s and a sharp decline of 8.8% at 0.033 kg/s. At 310 m/s, efficiency reductions are smoother, likely due to the limited number of simulated points. These results confirm that, beyond the surge line, compressor performance deteriorates, and reductions in both pressure ratio and efficiency serve as indicators of global instability. When fins are introduced, a noticeable but moderate efficiency loss occurs relative to the no-finned configuration, although both follow similar trends. At the manufacturer’s surge condition for 370 m/s, efficiency decreases by approximately 2.7% in transient and 4.1% in steady-state simulations. This difference widens slightly beyond the surge line, reaching 3.5% and 5.6% reductions at a mass flow rate of 0.039 kg/s for transient and steady-state cases, respectively. At the lower 310 m/s speed line, the differences are smaller: the finned configuration shows efficiency reductions of 0.7% (transient) and 2% (steady-state) at the surge point, which further increase to 1.2% and 4.7% beyond it.

**Fig. 15:**
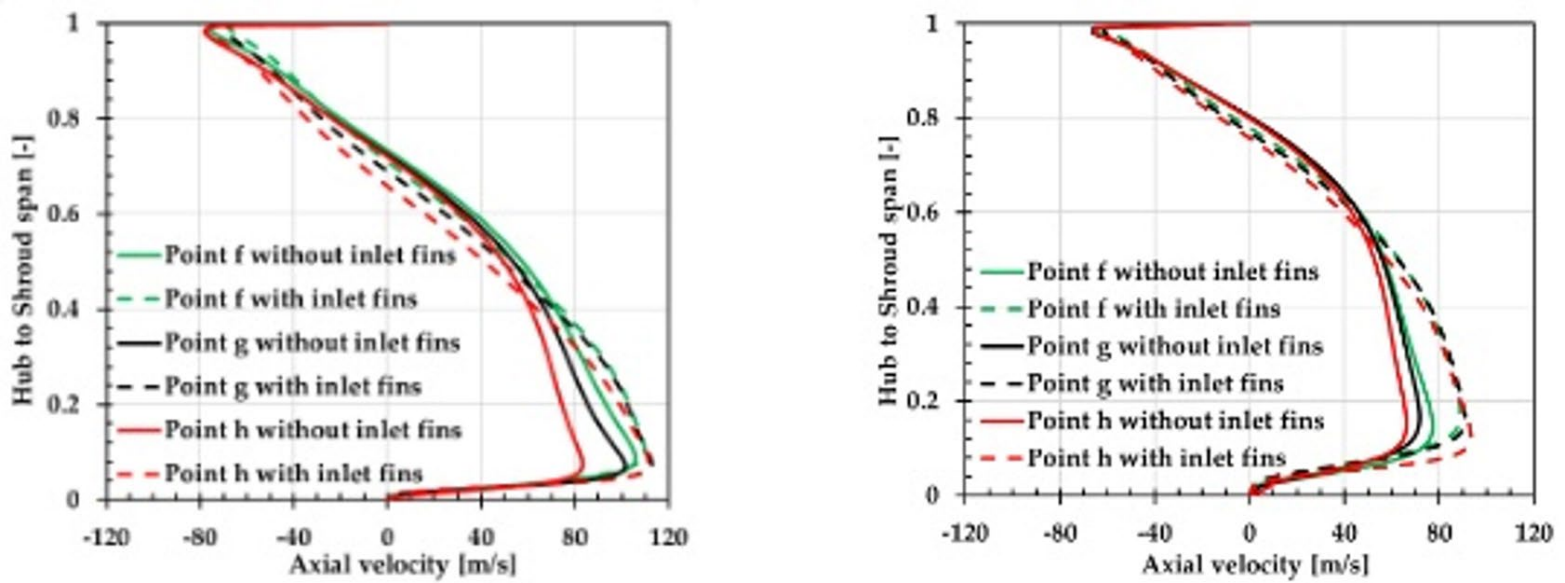
Axial velocity component profiles with inlet fins (Case F) and without inlet fins for the 370 m/s (left) and 310 m/s (right) speed lines (transient simulations; parameter was first time-averaged and then circumferentially averaged).

**Fig. 16:**
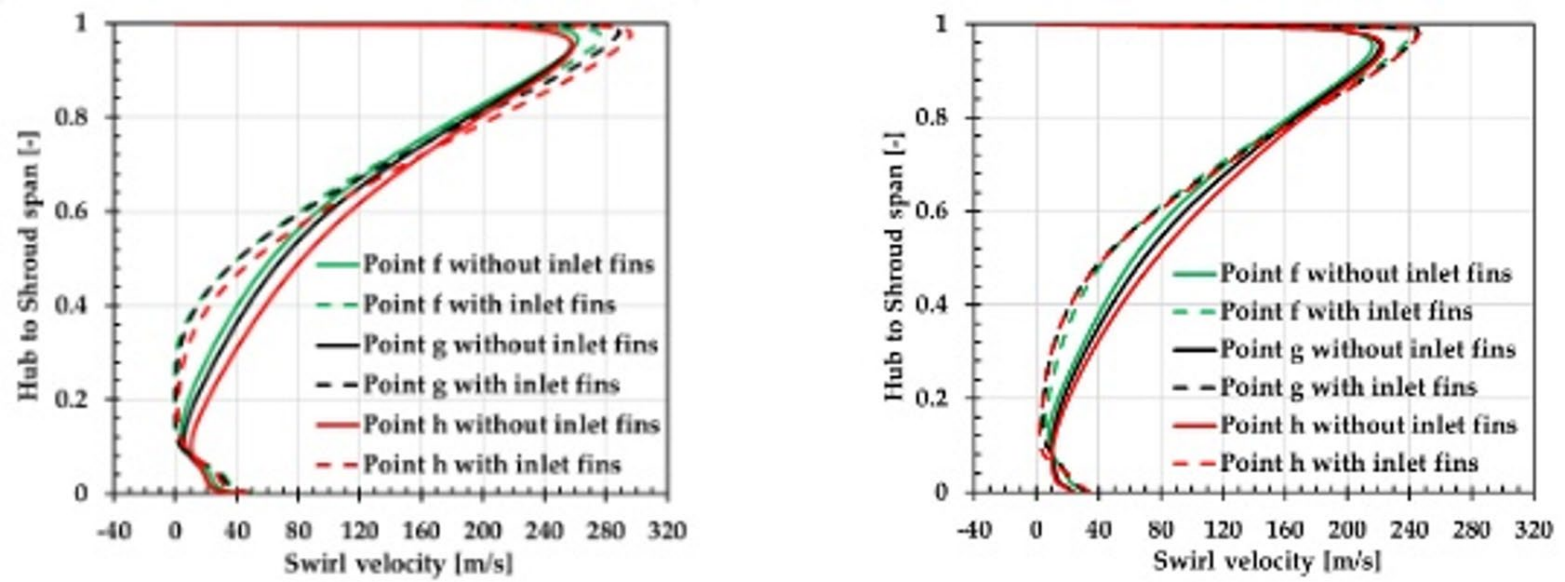
Circumferential velocity component profiles with inlet fins (Case F) and without inlet fins for the 370 m/s (left) and 310 m/s (right) speed lines (transient simulations; parameter was first time-averaged and then circumferentially averaged).

**Fig. 17:**
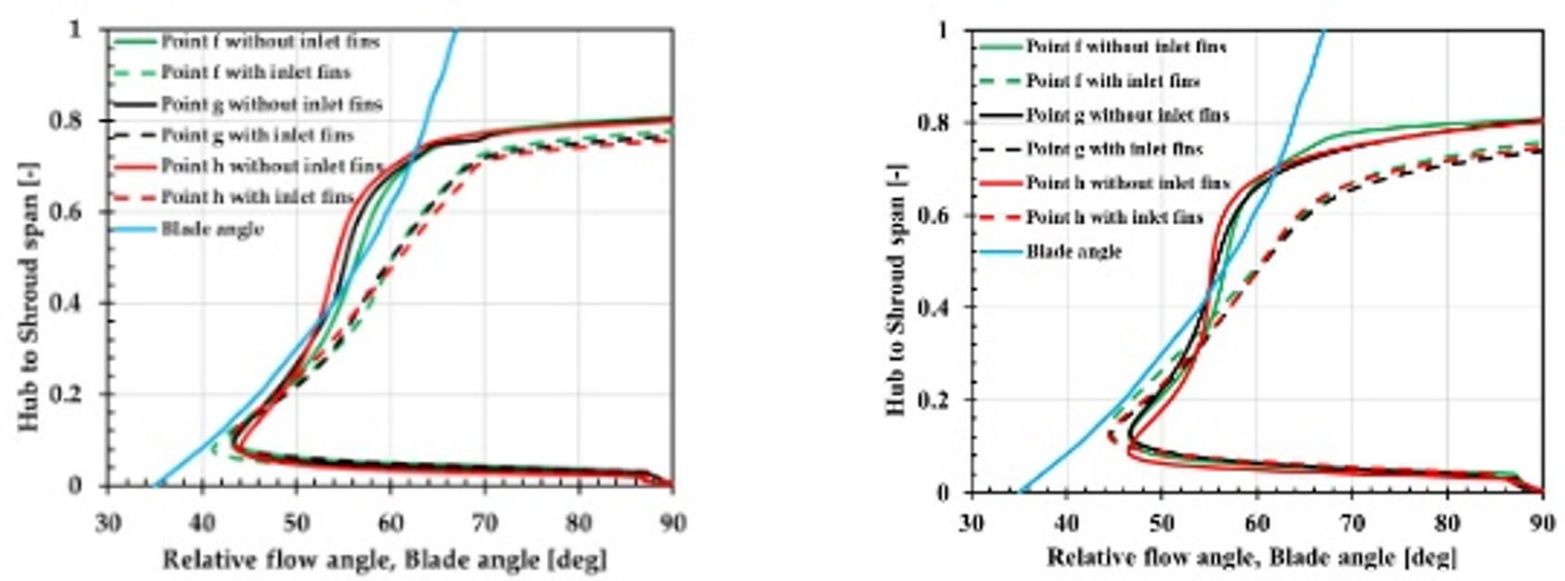
Relative flow angle and blade angle profiles with inlet fins (Case F) and without inlet fins for the 370 m/s (left) and 310 m/s (right) speed lines (transient simulations; parameter was first time-averaged and then circumferentially averaged).

One of the main factors contributing to the efficiency reduction is the increase in the incidence angle at the impeller leading edge. This effect results from the decreased preswirl caused by the interaction between the inlet fins and the incoming flow. A higher incidence angle promotes flow separation and elevates shear stresses on the blade surface. In addition, the inlet fins disturb the swirl flow and introduce additional dissipation and blockage effects in the inlet region, which increase total pressure losses and reduce overall efficiency. To illustrate this, Figs. 15, 16 and 17 present the axial and circumferential (swirl) velocity components, as well as the relative flow angles at the impeller inlet control plane, for both speed lines (370 m/s and 310 m/s) and configurations with and without fins (Case F). The results are based on circumferentially averaged, time-averaged data from transient simulations, corresponding to operating points (f), (g), and (h) identified in Fig. 12. Point (f) represents the manufacturer’s surge line, (g) marks the condition of maximum pressure ratio for the finned case, and (h) corresponds to a deteriorated performance state.

Across all operating points, significant spanwise variations in velocity distributions are observed depending on fin configuration and flow rate. In the finned cases, the axial velocity component increases up to 50% of the span compared to the no-finned setup, despite the reduced mass flow rate at both speed lines. For points (f) and (g), the distributions are nearly identical, while at point (h), a drop in mass flow results in lower axial velocity. Additionally, between 50 and 90% of the span, the finned configurations exhibit a marked velocity reduction, and the reverse flow zone extends further toward the shroud as the flow rate decreases. This effect is especially pronounced at 370 m/s, where the reverse flow boundary reaches 65% of the span for the finned configuration versus 72% for the no-finned one, with both showing comparable peak reverse velocities near the shroud.

As shown in Fig. 16, the preswirl effect is evident in all no-finned cases, where the swirl velocity remains positive over most of the span and intensifies with decreasing mass flow rate. The addition of fins reduces swirl to nearly zero up to 30–40% of the span and maintains lower swirl values than the no-finned configuration up to about 70–80%. Only near the shroud does the finned case exhibit slightly higher swirl. For point (h), further flow reduction causes stronger adverse preswirl effects.

Figure 17 demonstrates that, for both speed lines, the no-finned configuration exhibits progressively decreasing relative flow angles from point (f) to (h). This behavior arises because points (g) and (h) operate beyond the surge line, where intense inlet recirculation increases preswirl and reduces the incidence angle, between 40 and 70% of the span. As a result, the pressure ratio and efficiency drop significantly, as seen in Figs. 13 and 14. In contrast, the use of fins suppresses preswirl and maintains higher incidence angles (20–80% span). The finned configurations show nearly identical relative flow angle profiles across all three operating points, indicating stable inlet conditions and reduced upstream recirculation. Up to point (g), mass flow reduction has minimal impact on flow structure, but at point (h), significant changes in axial and swirl velocities indicate degraded flow conditions, explaining the pressure ratio and efficiency losses associated with early surge.

## Conclusions

The growing demand for efficient, low-emission vehicles has driven the development of downsized, turbocharged internal combustion engines. However, their wide operating range introduces challenges for compressor stability, particularly under low-flow conditions where phenomena such as inlet recirculation and surge significantly affect performance. This research combined experimental testing and high-fidelity CFD simulations to examine the suppression of inlet recirculation in a small turbocharger centrifugal compressor. The validated numerical models closely matched experimental data, capturing the complex unsteady flow structures that emerge near surge. A passive flow control approach using inlet fins was developed and tested. Optimally positioned fins near the impeller inlet effectively suppressed recirculation, reduced swirl intensity, and extended the compressor stable operating range.

The key findings of the present study are summarized below:

- Integration of inlet fins produced negligible changes in pressure ratio and isentropic efficiency at design conditions, confirming that they do not compromise performance within the stable operating range.
- At the off-design conditions, both the experimental and numerical results show that finned configurations achieved higher pressure ratios up to 1.5% and 5.4%, respectively, compared to the no-finned configuration.
- Numerical study demonstrates that inlet fins have a significant impact on the operating range and stability of the compressor. The application of inlet fins shifted the surge mass flow rate by up to 10.4% for the 370 m/s speed line and 8.4% for the 310 m/s speed line in transient simulations, indicating a substantial extension of the stable operating range, thereby reducing the risk of surge.
- Inlet fins suppress swirl across most of the span, improving flow stability, although their presence also reduces efficiency under near-surge conditions by up to approximately 5% in experiments at low flow rates.
- The decline in efficiency with inlet fins is attributed to an increased incidence angle at the impeller leading edge, caused by reduced preswirl due to fin–inlet flow interaction, which in turn promotes local flow separation.
- Despite a modest efficiency penalty being observed, the study confirms the practicality of fins as a simple and low-cost stabilization method of enhancing compressor performance and maintaining a stable operating range.

Future work should focus on high-resolution experimental measurements, enhanced CFD modeling using different boundary conditions and integration of recirculation-based monitoring into predictive surge control systems. In addition, further parametric study of the inlet fin design will be pursued to reduce efficiency losses and enhance overall flow stability and performance. The possibility of their optimization would be explored with GPU solvers.

## Data Availability

The model of the turbocharger compressor analyzed in this study is the property of BorgWarner and Lodz University of Technology and is therefore not publicly available. The model could be shared by the corresponding authors to the third party upon a reasonable request and with permission of BorgWaner Poland.

## Latin variables

$\:\dot{m}$: mass flow rate [kg/s]
*n*: rotational speed [rpm]
*p*: pressure [Pa]
*T*: temperature [K]
y+: dimensionless near-wall distance [–]

## Greek variables

$$\eta$$: efficiency [–]
$\gamma$: specific heat ratio [–]
$$\pi$$: pressure ratio [–]

## Subscripts

1: compressor inlet position
2: compressor outlet position
*is*: isentropic
*s*: surge
*t*: total
*tt*: total-to-total

## Abbreviations

3D: Three-dimensional
BC: Boundary conditions
CAD: Computer-aided design
CFD: Computational fluid dynamics
GCI: Grid convergence index
GGI: General grid interface
ICE: Internal combustion engine
IR: Inlet recirculation
LE: Leading edge
RANS: Reynolds averaged Navier–Stokes
RE: Richardson Extrapolation
RMS: Root mean square
SST: Shear stress transport
